# Multiomics reveal non-alcoholic fatty liver disease in rats following chronic exposure to an ultra-low dose of Roundup herbicide

**DOI:** 10.1038/srep39328

**Published:** 2017-01-09

**Authors:** Robin Mesnage, George Renney, Gilles-Eric Séralini, Malcolm Ward, Michael N. Antoniou

**Affiliations:** 1 Gene Expression and Therapy Group, King’s College London, Faculty of Life Sciences & Medicine, Department of Medical and Molecular Genetics, 8th Floor, Tower Wing, Guy’s Hospital, Great Maze Pond, London SE1 9RT, United Kingdom; 2 Proteomics Facility, King’s College London, Institute of Psychiatry, London SE5 8AF, United Kingdom; 3 University of Caen, Institute of Biology, EA 2608 and Risk Pole, MRSH-CNRS, Esplanade de la Paix, University of Caen, Caen 14032, Cedex, France

## Abstract

The impairment of liver function by low environmentally relevant doses of glyphosate-based herbicides (GBH) is still a debatable and unresolved matter. Previously we have shown that rats administered for 2 years with 0.1 ppb (50 ng/L glyphosate equivalent dilution; 4 ng/kg body weight/day daily intake) of a Roundup GBH formulation showed signs of enhanced liver injury as indicated by anatomorphological, blood/urine biochemical changes and transcriptome profiling. Here we present a multiomic study combining metabolome and proteome liver analyses to obtain further insight into the Roundup-induced pathology. Proteins significantly disturbed (214 out of 1906 detected, q < 0.05) were involved in organonitrogen metabolism and fatty acid β-oxidation. Proteome disturbances reflected peroxisomal proliferation, steatosis and necrosis. The metabolome analysis (55 metabolites altered out of 673 detected, p < 0.05) confirmed lipotoxic conditions and oxidative stress by showing an activation of glutathione and ascorbate free radical scavenger systems. Additionally, we found metabolite alterations associated with hallmarks of hepatotoxicity such as γ-glutamyl dipeptides, acylcarnitines, and proline derivatives. Overall, metabolome and proteome disturbances showed a substantial overlap with biomarkers of non-alcoholic fatty liver disease and its progression to steatohepatosis and thus confirm liver functional dysfunction resulting from chronic ultra-low dose GBH exposure.

Glyphosate-based herbicides (GBH), such as Roundup, are the major pesticides used worldwide^1^. Residues of GBH are routinely detected in foodstuffs^2^,^3^ and drinking water^4^. Epidemiological data on the human body burden of GBH residues is very limited but evidence suggests that glyphosate and its metabolites are widespread^5^. The active principle of GBH, glyphosate, is a competitive inhibitor of phosphoenolpyruvate^6^. Glyphosate acts as a herbicide by inhibiting 5-enolpyruvylshikimate-3-phosphate synthase (EPSPS) of the shikimate aromatic amino acid biosynthesis pathway present in plants and some bacteria^7^.

A number of toxicity studies have shown that glyphosate and its commercial formulations have non-target effects on mammalian metabolism and provoke toxic effects, especially with respect to liver and kidney structure and function^8^,^9^. Potential adverse hepatic effects of glyphosate were first observed in the 1980s, including its ability to disrupt liver mitochondrial oxidative phosphorylation^10^. As glyphosate can act as a protonophore increasing mitochondrial membrane permeability to protons and Ca^2+ ^11, it can trigger the production of reactive oxygen species resulting in observed oxidative stress^12^. Elevation in oxidative stress markers is detected in rat liver and kidney after subchronic exposure to GBH at the United States permitted glyphosate concentration of 700 μg/L in drinking water^13^. Hepatic histological changes and alterations of clinical biochemistry are detected in rats consuming 4.87 mg/kg body weight (bw) glyphosate every 2 days over 75 days^14^. In farm animals, elevated glyphosate urinary levels are correlated with alterations in blood serum parameters indicative of liver and kidney oxidative stress and depletion in nutrient trace element levels^15^.

Nevertheless, it should be noted that most results from these GBH toxicity studies were obtained at doses far greater than general human population exposure. Doses tested were typically over the glyphosate acceptable daily intake (ADI), which is currently set at 0.3 mg/kg bw/day within the European Union (1.75 mg/kg bw/day in the USA) based on hepatorenal toxicity measurements after chronic exposure in rats^16^,^17^. However, no long-term studies investigating the toxicity of complete GBH commercial formulations, which contain a broad spectrum of largely undisclosed “adjuvants” as well as glyphosate, have been conducted (see ref. 9). In an effort to address this gap in commercial GBH toxicity evaluation, a 2-year study was conducted where rats were administered with a Roundup GBH via drinking water at a concentration of 0.1 ppb (0.05 μg/L glyphosate; daily intake 4 ng/kg bw/day), which is an admissible concentration within the European Union (0.1 μg/L) and USA (700 μg/L)^18^. The results showed that Roundup caused an increased incidence in signs of anatomical pathologies, as well as changes in urine and blood biochemical parameters suggestive of liver and kidney functional insufficiency^18^.

Most pesticides exert their toxic effects by targeting proteins and modulating their activity. Herbicides act mostly by inhibiting plant enzymes responsible of photosynthesis, carotenoid synthesis, or amino acid synthesis^19^. Besides its well known interaction with EPSPS, it has been suggested that glyphosate could impact mitochondrial function by inhibiting succinate dehydrogenase^20^, or on steroid biosynthesis by inhibiting aromatase enzyme activity^21^. Molecular profiling techniques can be used to identify specific signatures of chemical toxicity and thus provide greater insight into organ pathological status^22^,^23^. The proteome and metabolome are very sensitive to toxic chemical exposures and have been used to reveal non-targets effects of herbicides such as paraquat^24^, atrazine^22^ and organophosphate mixtures^25^ in mammalian species. However, while transcriptome profiles reveal pathway disturbances that could be correlated to toxic effects, they do not always translate into alterations in protein levels and functional, metabolic disturbances. Overall, mRNA transcript abundance explains approximately one- to two-thirds of the variance in steady-state protein levels^26^. In yeast subjected to oxidative stress, a post-transcriptional regulation of a large fraction of the genes was observed independently of their up- or downregulation^27^.

Given the insight molecular profiling methods can potentially provide into processes and mechanisms of toxicity, we have previously conducted a transcriptomics investigation of the same female cohort of animals subjected to ultra-low dose Roundup exposure, and which showed signs of liver and kidney damage at a anatomorphological and blood/urine biochemical level of function^28^. Our previous results showed alterations in the liver transcriptome reflective of fibrosis, necrosis, phospholipidosis, mitochondrial membrane dysfunction and ischemia^29^. However, as changes in the transcriptome may not fully translate into alterations in organ function, we hypothesized that a study of the proteome and metabolome of the same liver tissues will provide confirmation of, and possibly a mechanistic link to, the type of liver pathology developed by rats exposed to Roundup. Our results show that proteins whose levels were altered were reflective of oxidative stress and changes in fatty acid metabolism. Proteome alterations were typical of disturbances measured in cases of peroxisomal proliferation, steatosis and necrosis. Metabolome analysis confirmed the induction of oxidative stress, and revealed hallmarks of hepatotoxicity. Overall, metabolome and proteome disturbances showed a substantial overlap with biomarkers of non-alcoholic fatty liver disease (NAFLD) and thus confirm metabolic dysfunction resulting from chronic exposure to an ultra-low dose of Roundup.

## Results

**Figure 1:**
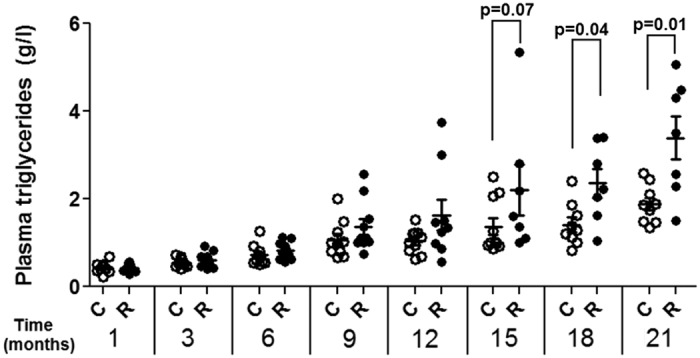
Increased plasma triglyceride levels in rats administered with Roundup. Blood samples was collected from control and Roundup-treated (50 ng/L glyphosate equivalent dilution) female rats following two years exposure via drinking water. Blood was taken via the tail vein of each animal after 1, 2, 3, 6, 9, 12, 15, 18, 21 and 24 months of treatment. Pair-wise comparisons were performed with a Mann-Whitney U test.

The female rat liver tissues, which formed the starting material for this investigation, were as previously described^29^. They were obtained from animals that formed part of a 2 year study of Roundup toxicity^18^. Harlan Sprague–Dawley rats were administered with Roundup via drinking water at a regulatory admissible dose (50 ng/L glyphosate). The average daily intake of Roundup was approximately 4 ng/kg bw/day glyphosate equivalent dose. Control and Roundup-treated animals were respectively euthanized at 701+/− 62 and 635+/− 131 days. Anatomopathological analysis of organs from these animals revealed that the liver was the most affected organ^18^. Roundup-treated female rats showed 3 times more anatomical signs of pathology (15 in 8 rats) than the control group (6 in 4 rats)^18^. Blood samples were collected from the tail vein of each rat under short isoflurane anesthesia after 1, 2, 3, 6, 9, 12, 15, 18, 21 and 24 months of treatment. Serum biochemical analysis showed increased levels of serum triglycerides (Fig. 1). Thus, although no differences were observed during the first year of the experiment, the rats administered with Roundup started progressively to accumulate serum triglycerides as they aged.

**Figure 2:**
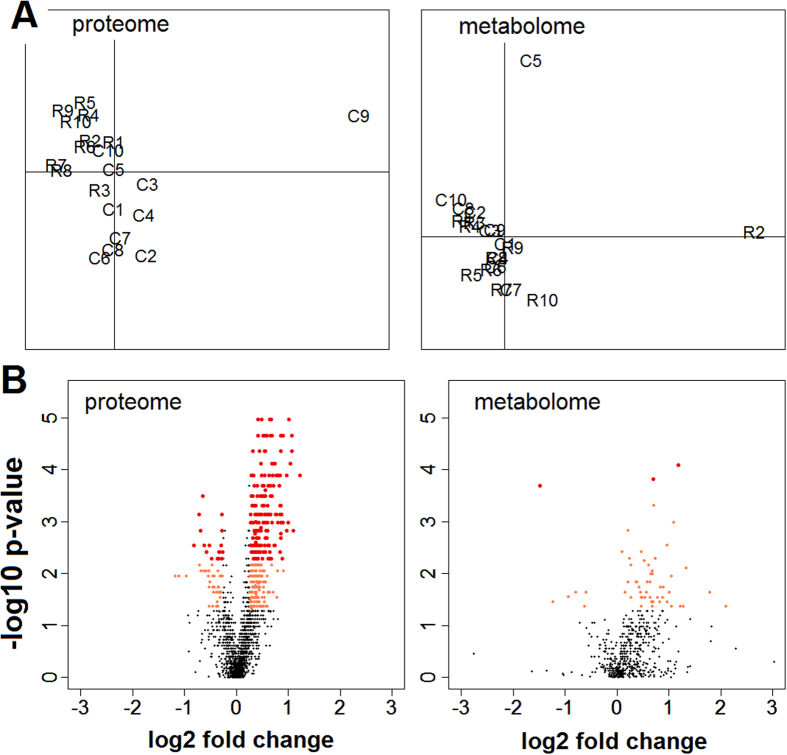
Wide-scale proteome and metabolome profile alteration in liver of Roundup-treated female rats. Liver from control rats and animals receiving 0.1 ppb Roundup (50 ng/L glyphosate equivalent dilution; 4 ng/kg body weight/day daily intake) in drinking water were subjected to a proteome and a metabolome analysis. (**A**) PCA analysis profiles shows a separation into groups of Roundup-treated (R) and control (C) rats in liver samples for the proteome but not for the metabolome analysis. (**B**) Volcano plots of liver profiles showing log 2 fold changes and the −log10 p-values in peptide and metabolite levels induced by Roundup exposure compared to controls. Data were used at the cut off values Benjamini-Hochberg adjusted p < 0.05 (dark red dots) or unadjusted p < 0.05 (light red dots), with a fold change >1.2.

The proteome discovery study consisted of a comparison between control (n = 10) and Roundup treated (n = 10) rat liver samples. Fractions for both non-enriched peptide and enriched phosphopeptides were analysed using Orbitrap Velos-Pro. A total of 1906 peptides were quantified across all liver samples. We began our analysis by looking at the variance structure in an unsupervised Principal Component Analysis (PCA). While percentages of explained variance on the 2 first components were low (22.3% and 14.3% respectively), a separation was observed between control and Roundup-treated rats (Fig. 2A). Of the 1906 quantifiable peptides taken forward for bioanalytical analysis, 214 were respectively found to be significantly regulated (Supplementary Table 3) at the cut off Benjamini-Hochberg adjusted p-value (or q-value) of 0.05 with a fold change (FC) of >1.2. Figure 2B shows the statistical significance of differential protein expression by volcano plot along with their respective FC.

**Figure 3:**
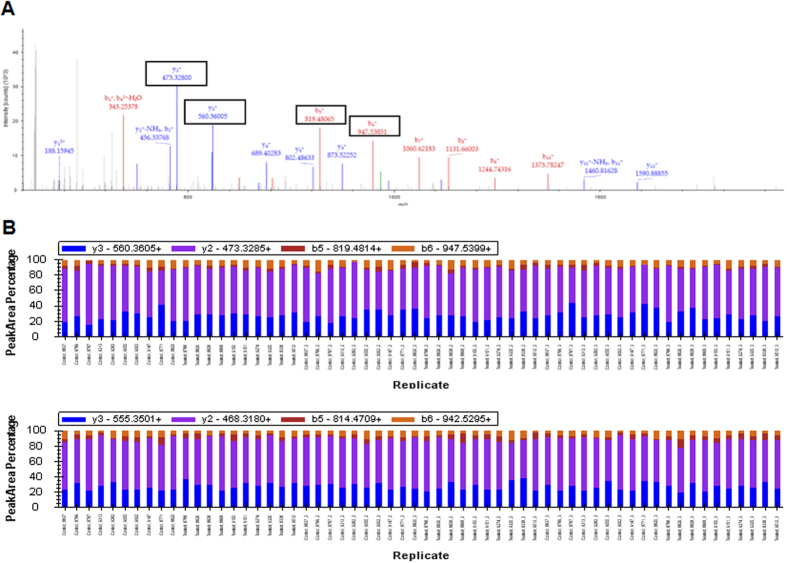
The raw LC-MS/MS spectra of 60S ribosomal protein L18. The peptide “ILTFDQLALESPK” from 60S ribosomal protein L18 detected in the discovery analysis via the Orbitrap Velos-Pro is presented along with the same peptides Transition Peak Area Percentage detection from TMT-SRM analysis via the TSQ Vantage.

**Figure 4:**
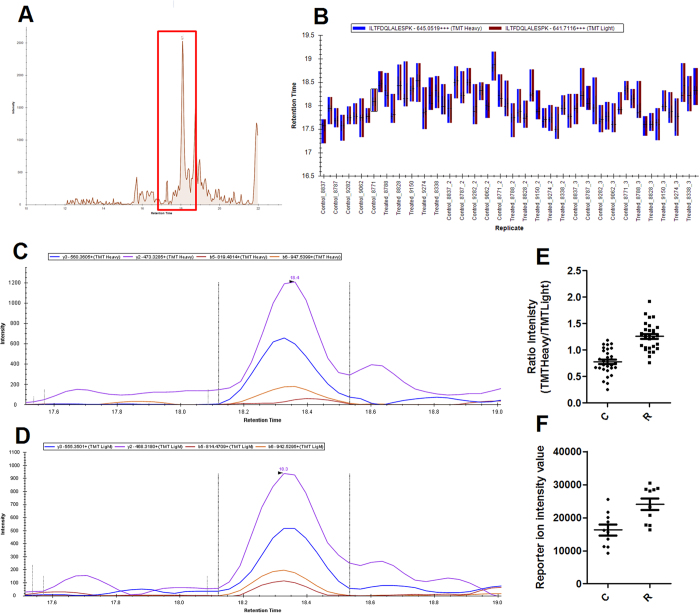
SRM verification of peptide “ILTFDQLALESPK” from 60S ribosomal protein L18. The TMT Heavy peptide transitions in the internal standard can be measured at the same time as the TMT Light peptide transitions in the sample of interest using the TSQ Vantage. The peak area under the transitions curves for the Light & Heavy transitions is then calculated to give a ratio specific to each sample. These are then compared between control and treated samples. This ratio change between control and treated samples can be compared in the same way that the reporter ion intensity values are in the discovery experiment. (**A**) Chromatogram of the sample run. (**B**) Box plot showing the retention times for each of the individual 60 samples. Co-eluting light (**C**) and heavy transition (**D**) in a control sample from a TSQ Vantage raw file analysed using Skyline software. Dot plots of comparisons between the SRM validation study (**E**) and the discovery study (**F**).

We developed a high throughput Tandem Mass Tag - Selected Reaction Monitoring (TMT-SRM) method to verify the alterations observed in protein levels in liver of Roundup-treated rats. The raw discovery LC-MS/MS spectra from Orbitrap Velos-Pro were used to select transition ions for each peptide (Fig. 3). First, in order to determine a subset of peptides, which can be detected by SRM, individual liver samples marked by a heavy TMT were combined with an internal standard constituted by all 20 samples marked by a light TMT. This method was repeated 4 times and any peptides/transitions, which were not detected in all 4 repeats removed. A total of 9 proteins and 10 peptides have been analysed over a 35 minute gradient. Then, 20 combined (TMTLight and TMTHeavy) liver samples were analysed on the TSQ Vantage in triplicate, leading to the production of 60 raw data files. The average CV value between technical repeats was 13.8%. Of the 10 peptides we aimed to quantify, 5 peptides were successfully detected across all 60 replicates. Several light and heavy transitions co-eluting at the same retention time were clearly identified, as illustrated for the peptide ‘ILTFDQLALESPK’ of the 60S ribosomal protein L18 (Fig. 4). All of 5 peptides showed a corresponding FC between the two methods (Table 1). The SRM data therefore corroborate protein alterations detected in the initial discovery analysis.

In order to obtain insight into the biological significance of the alterations in the proteome profile observed between the control and Roundup treatments groups, we next conducted an enrichment analysis integrating annotations from KEGG and Gene Ontology (GO) databases using DAVID 6.8 (Supplementary Table 5). The most enriched biological process was the response to drug (GO:0042493, FE = 6.0, BH corrected p = 1.9E^−8^), clearly indicating that the liver proteome of the treated animals are reflective of the treatment with a chemical agent (Table 2). Interestingly, the response to organonitrogen compounds (FE = 9.9, BH corrected p = 4.0E^−2^) was particularly enriched. Since glyphosate is an organonitrogen, this first set of proteins (BHMT, CYP2C7, HMGCS1, CYP2E1, MGST1) could constitute a signature of glyphosate metabolic effects. This is consistent with the glyphosate metabolic process (GO:0018920), which is defined as a type organonitrogen compound metabolic process (GO:1901564) in the GO database. A majority of the enriched terms are related to a modification of redox status (GO:0055114, FE = 4.9, BH corrected p = 2.1E^−7^). Overall, the proteome is reflective of an oxidative stress response (GO:0006979, FE = 8.5, BH corrected p = 7.6E^−4^). The alteration of glutathione metabolism (GO:0006749, FE = 21.1, BH corrected p = 1.1E^−5^) is indicative that the liver of the Roundup treated animals are likely to have suffered from an increase in oxidative stress.

**Figure 5:**
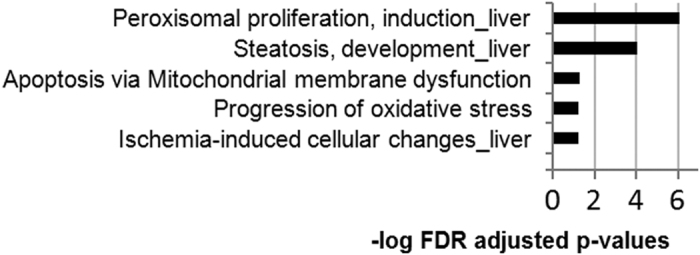
Toxicity ontology analysis of proteins disturbed in liver of Roundup-treated rats. List of top 5 scoring pathway and toxicity process networks revealed by MetaCore analysis of female liver proteome profiles receiving 0.1 ppb of Roundup in drinking water (q < 0.05, p < 0.005, fold changes >1.2). The p-values are determined by hyper-geometric calculation and adjusted using the Benjamini-Hochberg method.

A major significant cluster of annotation was related to fatty acid degradation (rno00071, FE = 11.5, BH corrected p = 9.1E^−5^). Of particular note is the modulation of triglyceride metabolism (GO:0006641, FE = 21.4, BH corrected p = 6.6E^−4^), which corroborates with the accumulation of serum triglycerides measured in these rats (Fig. 1). Another enriched GO term is indicative of the modulation of epoxygenase P450 pathways (GO:0019373, FE = 27.3, BH corrected p = 1.7E^−3^). Some P450 cytochromes had their levels altered by the Roundup treatment, namely CYP2C12, CYP2C7, CYP2C70, CYP2A1, CYP2D1, CYP2C6v1, CYP2D26 and CYP2E1, with all being overexpressed. This reflects a response to a xenobiotic stimulus by the modulation of detoxification pathways. The toxicity processes analysis in Metacore (Fig. 5) confirms lipotoxic conditions as revealed by disturbances in protein expression associated with the induction of peroxisomal proliferation (n = 14, BH corrected p = 8.8E^−7^) and steatosis (n = 16, BH corrected p = 9.1E^−5^). Collectively, these gene ontology terms are indicative of a change in lipid metabolism provoked by intoxication with the Roundup herbicide and imply the development of non-alcoholic fatty liver disease (NAFLD).

We next conducted a metabolome profiling of liver sections from the same animals to further investigate for the presence of markers of liver disease. Instrument variability determined by calculating the median relative standard deviation (RSD) for the internal standards was 6%. Overall process variability determined by calculating the median RSD for all endogenous metabolites was 12%. In total, 673 compounds were identified (Supplementary Table 8). By contrast to the results at the proteome level, the PCA of the metabolome samples (Fig. 2A) shows no clear separation of test and control groups. A volcano plot of statistical differences in metabolite levels is presented in Fig. 2B. Using the same statistical analytical methods we employed to analyse the proteomics dataset with a stringent statistical cut-off threshold (Benjamini Hochberg adjusted p ≤ 0.05), only 3 compounds (namely, N-methyl proline, N-acetyl-beta-alanine, nicotinamide riboside) were found to be significantly altered in the Roundup treated verses the control group (Supplementary Table 7). However, the 55 metabolites having a p-value < 0.05 were nevertheless considered for biological interpretation. Although the level of statistical significance of these 55 metabolites does not survive the multiple comparison tests, their biological interpretation provides a coherent explanation of the treatment effect due to the fact that these statistically significant differences were not randomly distributed among metabolic pathways but concentrated in those involved in the response to oxidative stress and lipotoxic conditions. Statistically significantly altered metabolites had FC values ranging from −2.76 (nicotinamide riboside) to 4.29 (2-hydroxyhippurate). Of the 55 metabolites having their levels changed, 12 had FC over 2. (Note: glyphosate (N-(phosphonomethyl) glycine) and its principal metabolite aminomethylphosphonic acid (AMPA) were not detected in liver tissue with a limit of detection of 7.8 ppb).

**Figure 6:**
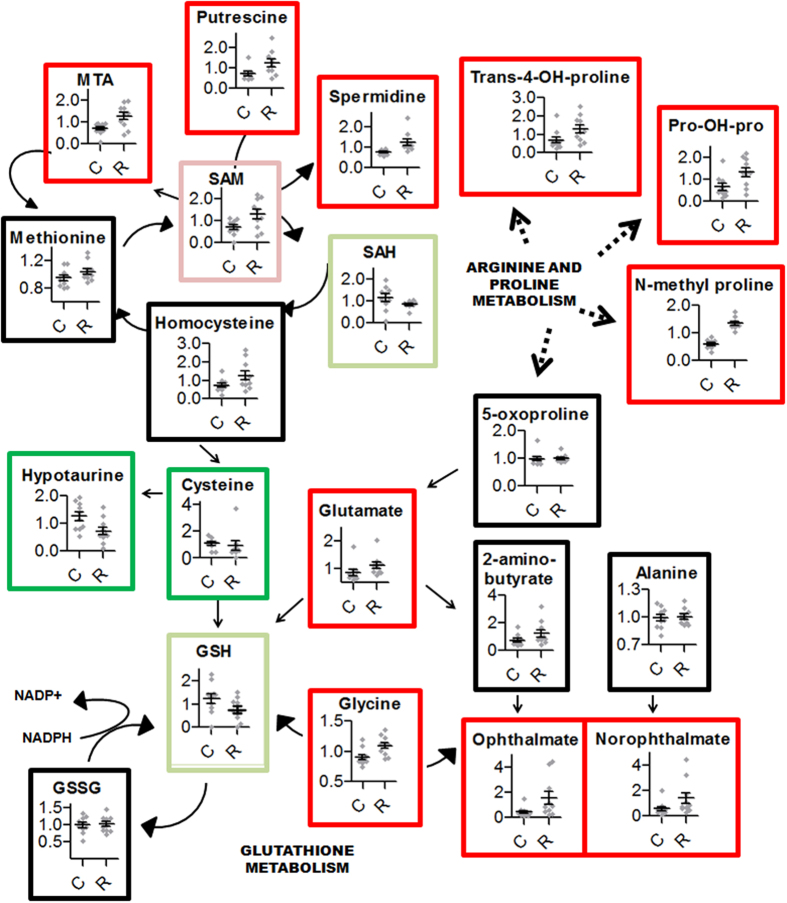
Scatter plots of the major significantly altered metabolic networks in livers of Roundup-treated female rats. Levels of each metabolite from the metabolomics of livers from female rats receiving the herbicide Roundup in their drinking water (R) were subjected to a statistical analysis by comparison to controls (C) using a Mann-Whitney U test. A selection of metabolites showing a statistically significant increase (dark red) or decrease (dark green) and which are of potential biological relevance are shown. Some metabolites approaching the level of significance (p < 0.1) are shown by light red and light green frames.

Biochemicals indicative of a change in cellular redox status were markedly disturbed (Table 3; Fig. 6). Ophthalmate (FC = 3.46, p = 0.02) and norophthalmate (FC = 2.42, p = 0.04) were significantly increased. They are endogenous analogue of glutathione (GSH) considered as markers for GSH depletion under oxidative stress conditions. A depletion of GSH was detected but did not reach statistical significance (FC = −1.64, p = 0.09). Gamma glutamyl dipeptides, indicators of the production of reduced glutathione, are increased in our study. Out of 10 gamma glutamyl dipeptides detected, 9 had their levels elevated (3 being significantly increased). Remarkably, cysteine levels (used to synthesize glutathione) were depleted (FC = −1.2, p = 0.07). Furthermore, because hypotaurine production is dependent on cysteine levels, its decrease (FC = −1.7, p = 0.02) suggests a redistribution of cellular cysteine stores toward glutathione synthesis that could deplete hypotaurine synthesis by substrate attrition. Cellular redox status disturbances are also corroborated by alterations in ascorbate metabolism (another intracellular free radical scavenger). Dehydroascorbate, ascorbate and oxalate levels were increased by the consumption of Roundup even if these did not reach statistical significance. Altogether, even though statistical significance was observed in only some cases, alterations in biochemical levels of the two major intracellular free radical scavenger systems consistently suggest cellular oxidative stress.

Higher levels of amino acids such as glycine (FC = 1.21 p = 0.007) and aspartate (FC = 1.50 p = 0.01), and of amino acid catabolites such as 2-aminoadipate (FC = 1.39 p = 0.01) were observed. In addition, many biochemicals associated with purine and pyrimidine metabolism were elevated, indicating heightened nucleic acid turnover. Levels of purine catabolites such as urate and allantoin, or pyrimidine catabolites such as β-alanine and 3-aminoisobutyrate, were either not detected or not altered, suggesting that an elevation in the levels of these compounds was indicative of nucleic acid turnover associated with growth rather than nucleic acid breakdown. The Roundup treatment group also presented an increased rate of polyamine synthesis, suggesting cell proliferation and organ regeneration. All of the 4 polyamines detected namely acisoga (FC = 1.95, p = 0.03), putrescine (FC = 1.77, p = 0.03), spermidine (FC = 1.67, p = 0.0005) and 5-methylthioadenosine (FC = 1.78, p = 0.02) were statistically significantly increased (Fig. 5). This was accompanied by an increased S-adenosylmethionine (SAM): S-adenosylhomocysteine (SAH) ratio, a regulator for the synthesis of spermidine. This biochemical signature most likely indicates that overall metabolism was higher in Roundup-treated animals.

Additionally, we found metabolite alterations associated hepatotoxicity biomarkers. Levels of proline derivatives were elevated in the liver of Roundup-treated rats (Fig. 5). In particular, N-methylproline a biomarker of fibrosis^30^ whose levels remained significant following multiple comparison adjustment, was increased by 2.27-fold (p = 0.00008) (Fig. 5). We also noticed alterations in nicotinate and nicotinamide metabolism intermediates. Levels of nicotinamide riboside (FC = −2.8, p = 0.0002) and nicotinamide ribonucleotide (FC = −2.3, p = 0.04) were decreased. Hepatic acylcarnitines, reflective of mitochondrial fatty acid oxidation impairment, were increased. We also observed an increased trend in hepatic accumulation of most medium and long-chain fatty acids as well as branched or dicarboxylate fatty acids. Furthermore, cholesterol levels were weakly disturbed (FC = 1.16, p = 0.002).

Collectively, alterations of the proteome and the metabolome profiles in the liver of rats chronically receiving an environmentally relevant concentration of Roundup in their drinking water show a substantial overlap with those characteristic of non-alcoholic fatty liver disease.

## Discussion

We report here the first *in vivo* multiomic analysis combining the proteome and metabolome profiles of the livers from rats following long-term (2-year) exposure to a GBH (Roundup) at an environmentally relevant dose (50 ng/L glyphosate equivalent concentration; 4 ng/kg bw/day). Our integrated analysis of these molecular profiles is clearly reflective of features of non-alcoholic fatty liver disease (NAFLD) and its progression to non-alcoholic steatohepatosis (NASH). Our study thus confirms the increased incidence of liver pathologies in these female rats, which was observed at an anatomorphological and blood/urine biochemical level^18^, and following transcriptome analysis^29^.

NASH results from a two-hit process^31^. The first hit is a metabolic disruption, marked by a hepatocellular accumulation of fatty acids, which then sensitizes the liver to further injury. Steatosis is associated with the accumulation of lipotoxic intermediates such as acylcarnitines. In the liver of rats administered with Roundup, a trend in the accumulation of most fatty acids, in particular acylcarnitines, as well as a statistically significant elevation of cholesterol levels was observed (Table 3). Proteome profiles further confirmed a marked change in lipid detoxifying metabolic processes (Table 1, Fig. 5). In the pathology of NASH, oxidative stress acts as a second hit^31^, leading to lipid peroxidation, mitochondrial damage, hepatocellular injury, and finally chronic inflammation and fibrosis. Proteome profiles were significantly enriched in features of peroxisomal proliferation, liver steatosis and necrosis. An association to features of NASH is further supported by enhancement of markers of oxidative stress and fibrosis in the metabolome profile (Fig. 5). The metabolomics analysis also indicates an increased cell proliferation. Tissue homeostasis relies on a delicate balance between apoptosis and cellular proliferation. When oxidative stress provokes apoptosis, injured cells are generally replaced through increased proliferation.

Our observations may have human health implication since NAFLD is predicted to be the next major global epidemic. Approximately 20–30% of the population in the United States carry extra fat in their livers^32^. NAFLD is associated with the recent rapid rise in the incidence of diabetes, obesity, and metabolic syndrome^33^. Overall, it is acknowledged that NAFLD is mostly caused by excess caloric intake, but also from the consumption of processed foods, which increases simple sugar and saturated fat ingestion as well as sedentary lifestyles^34^,^35^. However, many suffer from NAFLD but which do not have any high risk factors and thus other contributors to disease, such as exposure to physiologically active environmental pollutants via contaminated food, cannot be excluded. Recently, some endocrine disrupting chemicals have been implicated in the aetiology of metabolic syndrome. Commonly defined as obesogens, some environmental chemicals have been found to promote adipose cell differentiation and lipid storage in experimental animals and thus presumably also in humans^36^.

We detected metabolic alterations well below the glyphosate ADI (0.3 mg/kg bw/day) set within the European Union, and is within the range admitted in drinking water (0.1 ppb) and foodstuffs (for example, 20 ppm in soybeans or 2 ppm in bovine kidneys). However, inter-species biological relevance still needs to be ascertained because there is a lack of data pertaining to biological effects of glyphosate in human tissues at these levels to support or contradict our data.

The Roundup-induced liver pathologies confirmed in this report may arise from multiple sources as there is increasing evidence to suggest that GBHs and glyphosate can bring about toxic effects via different mechanisms, depending upon the level of exposure. Glyphosate has been suggested to act as an estrogen agonist based on assays in cultures of human breast cancer cells at comparable concentrations to the native hormone^37^,^38^. Other studies, albeit at much higher doses, have also shown that glyphosate can uncouple liver mitochondrial oxidative phosphorylation^10^. Glyphosate is also a patented antibiotic (Patent No.: US 7771736) and can inhibit the growth of susceptible bacteria by inhibition of the shikimate pathway and could cause dysbiosis in the gastrointestinal tract^39^. Additionally, because glyphosate herbicidal action results from competition for phosphoenolpyruvate (PEP) in plants, it is possible that similar effects are being exerted by this compound on other PEP utilizing enzymes including those in mammals. This could explain the effects of glyphosate on the activity of some TCA cycle enzymes observed in this study (Supplementary Table 3). In this regard the fact that glyphosate has been shown to interact with mitochondrial succinate dehydrogenase (SDH)^20^ is noteworthy. We hypothesize that glyphosate could interfere at multiple points within the TCA cycle by inhibiting enzymes having oxoacids as substrates.

One of the problems with the statistical analysis of the metabolome data we encountered was the lack of statistical significance following multiple comparison correction for all but three metabolites. However, the need for appropriate p-value adjustments is an important consideration to extract meaningful results from high-throughput experiments as conducted in this study^40^. Corrections for multiple comparisons reduce the chance of making type I errors (false positives) by increasing the chance of making type II errors (false negatives). In the case of metabolome data, p-value adjustments are calculated based on the number of metabolites detected in the experiment. Therefore, some biologically relevant biomarkers can have their statistical significance masked by the detection of a high number of unrelated metabolites as is the case in the study we present here. Overall, statistical significance should not be disconnected with biological interpretations but should also be balanced with the magnitude of the effect, the quality of the study and with findings from other studies^40^.

Although our results cannot provide insight into the mechanisms of the pathologies resulting from chronic very low–dose glyphosate exposure, they highlight the need for future GBH toxicity studies where organ molecular profiles are determined prior to appearance of the overt pathologies observed at late-stage termination as in this instance. Indeed, the use of aged animals may have enhanced variability. Additionally, differences in metabolite levels between individual animals within a group were greater than the effect of Roundup in some cases, which resulted in a lack of statistical power to survive multiple correction tests. However, in certain cases such as elevation in N-methylproline (Fig. 6), a biomarker of liver fibrosis^30^, remained statistically significant after multiple comparison adjustment. In addition, the non-adjusted statistically significant different changes in levels of metabolites such as trans-4-hydroxyproline and pro-hydroxy-pro were found to be non-random and thus biologically meaningful, and found to cluster in metabolic networks related to oxidative stress metabolism (Fig. 6). Nevertheless, larger cohorts would be needed to reach the statistical power required to reduce false discovery rates and increase the confidence in the conclusiveness of the small fold changes observed here. We emphasize that although the amplitude of the changes of certain metabolites was low, it is known that small changes over a long period of time can have major health consequences. For example, a 25% increase in serum HDL cholesterol levels is associated with reduced risk of death from ischaemic heart disease^41^.

An important consideration is that Roundup is not a single compound, but a mixture of an active ingredient (glyphosate) combined with various adjuvants, which are required to stabilise and allow penetration of glyphosate into plants. In short term acute exposures, some adjuvants can be considered as responsible of Roundup toxicity^42^. Populations of farmers exposed to adjuvants, such as solvents or petroleum distillates, possess a higher risk of developing hypospadias^43^ and present with more allergic and non-allergic wheeze conditions^44^. However, as adjuvant composition is proprietary and not fully disclosed, it is not possible to attribute the toxicity of the whole agricultural herbicide formulation to a given component. Future studies involving the administration of glyphosate alone would shed light on this issue.

## Conclusions

The results of the study presented here imply that chronic consumption of extremely low levels of a GBH formulation (Roundup), at admissible glyphosate-equivalent concentrations, are associated with marked alterations of the liver proteome and metabolome. These changes in molecular profile overlap substantially with biomarkers of NAFLD and its progression to NASH. These alterations correlate with the observed signs of hepatic anatomorphological and biochemical pathological changes in this organ^18^, and as suggested by transcriptome profiling^29^. Confirmatory studies incorporating testing principles from endocrinology should be performed to investigate potential implications of GBH low dose exposure in the development of metabolic syndrome.

## Methods

### Experimental design

The rat tissues analysed in this study were obtained from animals as previously described^18^. Briefly, the experimental protocol was as follows. Following 20 days of acclimatization, Harlan Sprague-Dawley rats at 5 weeks of age were randomly assigned on a weight basis into groups of 10 animals. Animals were fed with the standard diet A04 (Safe, France) including 33% maize DKC 2675 over two years. All feed formulations consisted of a balanced diet, chemically measured as substantially equivalent. All animals were kept in polycarbonate cages (820 cm^2^, Genestil, France). The location of each cage within the experimental room was regularly changed. The litter (Toplit classic, Safe, France) was replaced twice weekly. The animals were maintained at 22 ± 3 °C under controlled humidity (45% to 65%) and air purity with a 12 h-light/dark cycle, with free access to food and water. All reagents used were of analytical grade. The animal experimental protocol was conducted in accordance with the regulations of the local ethics committee in an animal care unit authorized by the French Ministries of Agriculture and Research (Agreement Number A35-288-1). Animal experiments were performed according to ethical guidelines of animal experimentation (regulation CEE 86/609).

Groups of 10 animals had access to either plain water (control) or to the same water supplemented with 1.1 × 10^−8^% of Roundup (0.1 ppb or 0.05 μg/L glyphosate equivalent dilution). The commercial formulation of Roundup used was Grand Travaux Plus (450 g/L glyphosate, approval 2020448; Monsanto, Belgium). The required level of Roundup dilution in drinking water was confirmed by measurement of glyphosate concentration by HPLC-MS/MS. Similarly, glyphosate stability in solution was studied and validated during the 7 day period between two preparations of the test treatment solutions.

Triglycerides were quantified by the enzymatic reactions of Fossati and Trinder^45^. The procedure measures the concentrations of total triglycerides by converting them to glycerol and free fatty acids by lipase. The glycerol is then converted to glycerol-3-phosphate and finally to hydrogen peroxide. A colored complex is formed from hydrogen peroxide, 4-aminophenazone and 4-chlorophenol via an enzymatic reaction with a peroxidase. The absorbance of the complex is measured at 510 nm.

### Tissue sampling

Animals were sacrificed at the same time of day during the course of the study either to comply with animal welfare regulations to avoid unnecessary suffering (for example, resulting from 25% body weight loss, presence of tumours over 25% bodyweight, hemorrhagic bleeding, or prostration) or at the termination of the study period of 2 years. Animals were sacrificed by exsanguination under isoflurane anesthesia. Livers were divided in two and half snap frozen in liquid nitrogen/dry ice and stored at −80 °C.

### Proteome profiling using Tandem Mass Tag-LC-MS/MS

Transverse cross sectional slices of liver were lysed in 8 M lysis buffer (urea, NaCl, Tri-HCl, dH2O, phosphatase and protease inhibitor) and the protein concentration of the resulting homogenate calculated using a Nanodrop protein assay (used on the A280 setting). Samples were reduced with 5 mM dithiotreitol (Sigma, UK), alkylated by treatment with 14 mM iodoacetamide (Sigma) and digested with 12 μg bovine sequencing grade trypsin (Roche, Germany, Ref. 11418475001) at 37 °C for 18 hours. Subsequently, peptides were purified and extracted using Waters Sep-Pak Vac 3cc 200 mg tC18 cartridges (Waters, WAT054925) in accordance to with the manufacturer’s instructions before each sample was labelled by incubation with 60 mM TMT10plex Isobaric Label Reagents (Thermo-Scientific, ref 90406). Labelled peptides were then purified and extracted again with the Waters Sep-Pak Vac 3cc 200 mg tC18 cartridge, before being fractionated by strong cation exchange (SCX) across an increasing salt concentration using elution buffers containing different concentrations of KCl: ranging from 0 mM KCl in the first fraction to 350 mM KCl in the 10th fraction. A 1/10^th^ aliquot of the eluted peptide fractions were separated and lyophilised for direct analysis by LC-MS/MS. The remaining 9/10^th^ of column eluate was enriched for phosphopeptides using a Pierce TiO_2_ Phosphopeptide Enrichment and Clean-up Kit (Pierce, Prod # 88301).

Un-enriched samples were re-suspended in 100 μl of 50 mM ammonium bicarbonate and F1&6, F2&7, F3&8, F4&9 and F5&10 pooled to give 5 fractions. Phosphopeptide enriched samples were re-suspended in 30 μl 50 mM ammonium bicarbonate and pooled in the same way as the un-enriched samples to also give 5 fractions. Both enriched (8 μl) and un-enriched (5 μl) fractions were loaded onto a 50 cm EASY-spray column (Thermo Scientific) and quantitative analysis was performed using the Orbitrap Velos-Pro mass spectrometer (Thermo Scientific) in positive ion mode. The peptides and phosphopeptides were separated by gradient elution, from 5–80% 0.1% trifluoroacetic acid in acetonitrile (5–40% from 0–100 minutes, 40–80% from 100–110 minutes), at a flow rate of 300 nl/min. Mass spectra (m/z) ranging from 400–1600 Daltons was acquired at a resolution of 60,000 and the 10 most intense ions were subjected to MS/MS by HCD fragmentation with 35% collision energy.

Protein identification was performed with Proteome Discoverer 1.4 (Thermo Fisher Scientific Inc.). Raw files were imported and searched against the UniProtKB/Swiss-Prot Database using Sequest for Proteome Discoverer. Exported raw data for the two TMT10plex sets is available as Supplementary Tables 1 and 2. Raw files for both enriched and un-enriched fractions were merged together in a single file search for each of the two TMT10plex sets. Precursor mass tolerance for the searches was set at 20ppm and fragment mass tolerance at 0.8ppm. The taxonomy selected was *Rattus norvegicus* and three enzymatic miscleavages were allowed. Dynamic modifications selected on the search were Oxidation/+15.995 Da (M), Phospho/+79.966 Da (S, T, Y) and Deamidated/+0.984 (N, Q) and static modifications were Carbamidomethyl/+57.021 Da (C), TMT10plex/229.163 Da (K), TMT10plex/229.163 Da (Any N-terminus).

### Proteome verification using Tandem Mass Tag - selected reaction monitoring experiment

Samples were prepared in the same way as the discovery experiment up to the TMT labelling stage. For selected reaction monitoring (SRM) analysis the twenty individually TMT10plex Heavy labelled samples were not combined but analysed individually against a pool of the twenty liver samples labelled with TMTZero/Light Label Reagent (Thermo-Scientific) included as a single point reference sample. All labelled peptides were purified and extracted before being lyophilised prior to mass spectrometry analysis.

Peptides were selected for TMT-SRM verification based on findings from the discovery analysis. Peptides chosen were significantly regulated against parameters where peptides had to display a fold change ≥1.2, either up or down-regulated, whilst being significant to a p-value of ≤0.01. Peptides were re-solubilised in 2% acetonitrile/0.1% trifluoroacetic acid and 2.5 μg of protein loaded onto a 50 cm EASY-Spray column (Thermo Scientific) using initial gradient conditions identical to those used in the discovery experiment. The LC system was coupled to a TSQ Vantage mass spectrometer (Thermo-Scientific) set in positive ion mode with Q1 and Q3 peak width settings of 1 full width at half its maximum height (FWHM). Capillary temperature (°C), collision gas pressure (mTorr) and spray voltage (V) were set at 270, 1.2 and 1800 respectively. In order to asses peptide transition specificity and abundance and define retention times, initial optimisation was performed using the reference sample alone. Following this a combined 1:1 mix with a random Heavy TMT labelled sample was then measured. The final method contained all successful transitions for both TMT Heavy and TMT Light versions of the peptides (Supplementary Table 4; 68 transitions from 10 peptides and 9 proteins). This final method was applied in three separate injections to each Heavy labelled experimental sample, which had been mixed in a 1:1 ratio with the Light labelled reference sample. Peptides were separated over 35 minutes, using gradient elution 5–80% 0.1% trifluoroacetic acid in acetonitrile (0–5% from 0–3 minutes, 5–50% from 3–7 minutes, 50–65% from 7–30 minutes and 65–80% from 30–35 minutes) at a flow rate of 300 nl/min.

Data was analysed using Skyline software^46^, with all peak matching also being visually verified. Peak area ratios between Light and Heavy transitions were generated for each sample and exported into Excel. When importing raw SRM data files from the TSQ vantage into Skyline software, a unique peak picking algorithm was used to confidently and accurately assign the best transition peaks for each peptide. Peptides were only taken forward for analysis if all assigned peaks were within a similar retention time period across all 60 samples. From the assigned peaks Skyline software was then used to produce a ‘total ratio’ between the Light and Heavy transitions for all of the 60 raw data files (3 × 10 control sample injection and 3 × 10 treated sample injections). This ‘total ratio’ value is a mean of the transition ratios, where the ratio is the comparison of the heavy transition peak areas to the light transition peak areas. The total ratio was averaged across all control and treated samples and a fold change between the two calculated. Coefficient of variation (CV) values between technical sample repeats were calculated and also averaged across control and treatment samples. Any peptides which displayed an average technical CV value higher than 15% were excluded from further analysis.

### Metabolome analysis

Semiquantitative metabolomics analysis was performed by ultra-high performance liquid chromatography-tandem mass spectroscopy (UPLC-MS/MS) and gas chromatography-mass spectroscopy (GC-MS) at Metabolon Inc. (Durham, NC, USA)^47^.

Samples were prepared using the automated MicroLab STAR® system from Hamilton Company (Reno, NV, USA). A recovery standard was added prior to the first step in the extraction process for QC purposes. In order to remove protein, dissociate small molecules bound to protein or trapped in the precipitated protein matrix, and to recover chemically diverse metabolites, proteins were precipitated with methanol under vigorous shaking for 2 min (Glen Mills GenoGrinder, 2000) followed by centrifugation. The resulting extract was divided into five fractions: one for analysis by UPLC-MS/MS with positive ion mode electrospray ionization, one for analysis by UPLC-MS/MS with negative ion mode electrospray ionization, one for LC polar platform, one for analysis by GC-MS, and one sample was reserved for backup. Samples were placed briefly on a TurboVap® (SOTAX Corp, Westborough, MA, USA) to remove the organic solvent. For LC, the samples were stored overnight under nitrogen before preparation for analysis. For GC, each sample was dried under vacuum overnight before preparation for analysis.

The LC-MS portion of the platform was based on a Waters ACQUITY ultra-performance liquid chromatography (UPLC) system (Waters Corp, Milford, MA, USA) and a ThermoFisher Scientific Q-Exactive high resolution/accurate mass orbitrap mass spectrometer operated at a 35,000 mass resolution, which was interfaced with a heated electrospray ionization (HESI) source. The sample extract was dried then reconstituted in acidic or basic LC-compatible solvents, each of which contained 12 or more injection standards at fixed concentrations to ensure injection and chromatographic consistency. One aliquot was analyzed using acidic positive ion-optimized conditions and the other using basic negative ion-optimized conditions in two independent injections using separate dedicated columns (Waters UPLC BEH C18-2.1 × 100 mm, 1.7 μm). Extracts reconstituted in acidic conditions were gradient eluted using water and methanol containing 0.1% formic acid, while the basic extracts, which also used water/methanol, contained 6.5 mM ammonium bicarbonate. A third aliquot was analyzed via negative ionization following elution from a HILIC column (Waters UPLC BEH Amide 2.1 × 150 mm, 1.7 μm) using a gradient consisting of water and acetonitrile with 10 mM ammonium formate. The MS analysis alternated between MS and data-dependent MS/MS scans using dynamic exclusion and the scan range was from 80–1000 m/z.

The samples destined for analysis by GC-MS were dried under vacuum for a minimum of 18 h prior to being derivatized under dried nitrogen using bistrimethyl-silyltrifluoroacetamide. Derivatized samples were separated on a 5% diphenyl/95% dimethyl polysiloxane fused silica column (20 m × 0.18 mm ID; 0.18 μm film thickness) with helium as carrier gas and a temperature ramp from 60° to 340 °C in a 17.5 minute period. Samples were analyzed on a Thermo-Finnigan Trace DSQ fast-scanning single-quadrupole mass spectrometer using electron impact ionization (EI) and operated at unit mass resolving power. The scan range was from 50–750 m/z.

### Bioinformatics analysis

For the proteome, only peptides with TMT reporter ion signal intensities for all ten samples were used for further bioinformatics analysis. Any duplicate peptides were removed before the data was SumScale normalised. The two normalised data files were then merged together to give one 10vs10 file comparison. Any peptides which did not have intensity values in all twenty TMT reporter ion channels were filtered out and median values were taken of the control and treated samples respectively. A total of 1906 peptides were quantified across all liver samples.

For the metabolome, raw data was extracted, peak-identified and QC processed using Metabolon’s hardware and software. Raw data is available as Supplementary Table 6. Metabolites were identified by automated comparison of the ion features in the experimental samples against a reference library of more than 3000 purified standard compounds that included retention time/index (RI), mass to charge ratio (m/z), and chromatographic data (including MS/MS spectral data), and then curated by visual inspection for quality control using software developed at Metabolon^48^. Peaks were quantified using area-under-the-curve. A total of 673 metabolites were detected. The maximum percent missing data allowed was 20%. As a result, 602 metabolites were taken forward for bioanalytical analysis.

The language and statistical environment R^49^ together with the ade4 package^50^ method was employed in order to explore the relationship between the control and the treated samples. We regressed out the batch effects between TMT1 and TMT2 from the protein expression data, and to correct variation resulting from instrument inter-day tuning differences for the metabolome data, using the limma package removeBatchEffect^51^. For plotting of results, a Principal Component Analysis (PCA) was first performed. Missing data was imputed by the value of the median of the group for a given metabolite in order to perform the PCA analysis. A previous evaluation of different fold change (FC) rules have found that a 1.2-fold change could be regarded as indicative of a significantly varying protein in TMT-LC-MS/MS experiments^52^. Data used for the functional analysis were selected at the cut off values of q < 0.05 (p < 0.005) with FC > 1.2. Pairwise non-parametric Mann–Whitney U tests were performed and a p-value was attributed to each of the 1906 peptides and the 602 metabolites. The resulting p-values were adjusted by the Benjamini-Hochberg multi-test adjustment method for a high number of comparisons. Volcano plots were also constructed in order to visualize the differences in metabolite and protein expression for each of the comparisons. The aforementioned tests and plots were performed using in-house R scripts. The pathway analysis was done using the Thomson Reuters MetaCore Analytical Suite and/or the NIH Database for Annotation, Visualization and Integrated Discovery Bioinformatics Resources 6.7 (DAVID) using recommended analytical parameters^53^.

## Additional Information

**How to cite this article**: Mesnage, R. *et al*. Multiomics reveal non-alcoholic fatty liver disease in rats following chronic exposure to an ultra-low dose of Roundup herbicide. *Sci. Rep.* **7**, 39328; doi: 10.1038/srep39328 (2017).

**Publisher's note:** Springer Nature remains neutral with regard to jurisdictional claims in published maps and institutional affiliations.

## Supplementary Material

Supplementary Data Legends

Supplementary Dataset 1

Supplementary Dataset 2

Supplementary Dataset 3

Supplementary Dataset 4

Supplementary Dataset 5

Supplementary Dataset 6

Supplementary Dataset 7

Supplementary Dataset 8

## Figures and Tables

**Table 1:** The discovery experiment is confirmed by SRM.

Peptide Sequence	Protein Descriptions	SRM			Disc. Exp.	
		FC	P	CV (%)	FC	P
aLTVPELTQQMFDAk	Tubulin beta-3 chain	1.60	5.3e-6	13.8	1.56	3.9e-3
eQFYHk	Hydroxymethylglutaryl-CoA synthase, mitochondrial	1.01	4.6e-1	13.8	1.40	6.8e-3
eTLMDLGTk	Carbamoyl-phosphate synthase [ammonia], mitochondrial	1.1	1.5e-3	7.5	1.40	1.1e-2
iLTFDQLALESPk	60S ribosomal protein L18	1.62	5.3e-5	13.1	1.47	2.2e-5
rFEYEDPFFNR	Cytochrome P450 2D26	1.14	8.4e-2	10.0	1.47	4.3e-5

Detected fold changes (FC) and p-values (P) of 5 of the 6 successfully analysed peptides through the SRM method are presented and compared to fold changes and p-values from the discovery experiment. The average technical repeat CV Value (%) (CV) from the SRM validation is indicated.

**Table 2:** Functional annotation analysis of liver genes using the DAVID tool.

GO terms in the Biological Process ontology	n	adj p-value	FE
GO:0042493~response to drug	23	1.9E-08	6.0
GO:0055114~oxidation-reduction process	24	2.1E-07	4.9
GO:0006457~protein folding	11	3.0E-06	12.9
GO:0071353~cellular response to interleukin-4	7	7.8E-06	34.2
GO:0006412~translation	16	1.3E-05	5.9
GO:0006749~glutathione metabolic process	8	1.1E-05	21.1
GO:0001889~liver development	10	9.9E-05	9.6
GO:0045471~response to ethanol	10	6.8E-04	7.5
GO:0006641~triglyceride metabolic process	6	6.6E-04	21.4
GO:0006979~response to oxidative stress	9	7.6E-04	8.5
**KEGG pathway annotation**	**n**	**adj p-value**	**FE**
rno01100:Metabolic pathways	55	1.4E-12	2.9
rno01130:Biosynthesis of antibiotics	19	1.7E-07	5.8
rno04141:Protein processing in endoplasmic reticulum	16	7.1E-07	6.5
rno01230:Biosynthesis of amino acids	12	9.4E-07	9.8
rno01200:Carbon metabolism	13	4.2E-06	7.3
rno03010:Ribosome	14	2.6E-05	5.5
rno00071:Fatty acid degradation	8	9.1E-05	11.5
rno00280:Valine, leucine and isoleucine degradation	8	2.3E-04	9.8
rno04146:Peroxisome	9	4.7E-04	7.1
rno00410:beta-Alanine metabolism	6	1.7E-03	11.9

The rat genome was used as a background list to calculate the p-values of each term. A total of 131 genes were recognised. The p-values were calculated according to a modified Fisher’s exact test (EASE score) and adjusted according to the Benjamini-Hochberg method. The fold enrichment (FE) of the statistically most overrepresented (enriched) GO biological process and KEGG pathway terms are presented.

**Table 3:** List of liver metabolites having their levels significantly altered by the Roundup treatment.

PUBCHEM ID	BIOCHEMICAL	FC	p-value	BH adj p-value
557	N-methyl proline	2.27	8.2E-05	4.1E-02
76406	N-acetyl-beta-alanine	1.62	1.5E-04	4.1E-02
439924	nicotinamide riboside	−2.79	2.1E-04	4.1E-02
1102	spermidine	1.64	4.9E-04	7.3E-02
439281	N-acetylmannosamine	2.13	1.1E-03	1.3E-01
11025495	cholesterol	1.16	1.5E-03	1.5E-01
5789	thymidine	1.96	2.9E-03	2.5E-01
13711	2′-deoxycytidine	1.39	3.9E-03	2.6E-01
9794176	gulonic acid	1.07	3.9E-03	2.6E-01
190	adenine	1.66	5.2E-03	2.8E-01
439197	N-acetylneuraminate	1.19	5.2E-03	2.8E-01
440129	imidazole lactate	1.44	5.7E-03	2.8E-01
0	chiro-inositol	1.53	6.8E-03	2.9E-01
750	glycine	1.21	6.8E-03	2.9E-01
NA	2,8-quinolinediol	2.52	7.8E-03	3.1E-01
69362	beta-hydroxyisovalerate	1.61	8.9E-03	3.4E-01
187790	2′-deoxyguanosine	1.58	1.0E-02	3.4E-01
65058	2′-deoxyinosine	1.61	1.0E-02	3.4E-01
3083879	catechol sulfate	2.08	1.1E-02	3.6E-01
5960	aspartate	1.50	1.5E-02	3.7E-01
123831	dimethylarginine (SDMA + ADMA)	1.16	1.5E-02	3.7E-01
151023	gamma-glutamylleucine	1.57	1.5E-02	3.7E-01
611	glutamate	1.31	1.5E-02	3.7E-01
151152	threonate	1.29	1.5E-02	3.7E-01
439176	5-methylthioadenosine (MTA)	1.78	1.9E-02	4.0E-01
68662	glutamate, gamma-methyl ester	1.35	1.9E-02	4.0E-01
5810	trans-4-hydroxyproline	1.85	1.9E-02	4.0E-01
64960	1,5-anhydroglucitol (1,5-AG)	1.38	2.3E-02	4.0E-01
9378	3-(4-hydroxyphenyl)lactate	−1.51	2.3E-02	4.0E-01
6267	asparagine	1.12	2.3E-02	4.0E-01
107812	hypotaurine	−1.74	2.3E-02	4.0E-01
0	methyl glucopyranoside (alpha + beta)	1.38	2.3E-02	4.0E-01
7018721	ophthalmate	3.47	2.3E-02	4.0E-01
11673055	pro-hydroxy-pro	2.01	2.3E-02	4.0E-01
1135	thymine	1.49	2.3E-02	4.0E-01
440364	N-acetyl-glucosamine 1-phosphate	−1.90	2.8E-02	4.0E-01
469	2-aminoadipate	1.39	2.9E-02	4.0E-01
128145	3-methylglutarylcarnitine (2)	1.85	2.9E-02	4.0E-01
440055	5-methyl-2′-deoxycytidine	1.21	2.9E-02	4.0E-01
6441454	docosapentaenoate (n6 DPA; 22:5n6)	1.72	2.9E-02	4.0E-01
65254;14284565	gamma-glutamyllysine	1.46	2.9E-02	4.0E-01
53481617	hydroxybutyrylcarnitine	1.57	2.9E-02	4.0E-01
92751	orotidine	1.38	2.9E-02	4.0E-01
129397	acisoga	1.95	3.5E-02	4.5E-01
3013625	glycylproline	1.60	3.5E-02	4.5E-01
14180	nicotinamide ribonucleotide (NMN)	−2.35	3.5E-02	4.5E-01
6602431	xylonate	1.62	3.5E-02	4.5E-01
1045	putrescine	1.77	3.6E-02	4.5E-01
65065	N-acetylaspartate (NAA)	2.08	4.3E-02	4.8E-01
70912	N-acetylleucine	1.61	4.3E-02	4.8E-01
5489007	norophthalmate	2.42	4.3E-02	4.8E-01
10253	2-hydroxyhippurate (salicylurate)	4.30	4.3E-02	4.8E-01
64956	3-aminoisobutyrate	1.38	4.3E-02	4.8E-01
444485	3′-dephosphocoenzyme A	−1.54	4.3E-02	4.8E-01
7019993	asparagylvaline	2.34	4.3E-02	4.8E-01
445408	5-methyluridine (ribothymidine)	1.61	5.2E-02	4.9E-01
2266	azelate (nonanedioate)	1.23	5.2E-02	4.9E-01
14253342	gamma-glutamylisoleucine	1.49	5.2E-02	4.9E-01
6426853	hexanoylcarnitine	1.50	5.2E-02	4.9E-01
2802421	N-acetylisoleucine	1.82	5.2E-02	4.9E-01
74839	N-acetylphenylalanine	1.82	5.2E-02	4.9E-01
152204	N-acetylthreonine	1.32	5.2E-02	4.9E-01
34755	S-adenosylmethionine (SAM)	1.84	5.2E-02	4.9E-01
1174	uracil	1.36	5.2E-02	4.9E-01

Levels of each metabolite from the metabolomics of livers from female rats receiving the herbicide Roundup in their drinking water were subjected to a statistical analysis by comparison to controls using a Mann–Whitney U test. A selection of metabolites showing a statistically significant change, as well as their fold changes and p-values is shown.
