# Author Correction: Multiomics reveal non-alcoholic fatty liver disease in rats following chronic exposure to an ultra-low dose of Roundup herbicide

**DOI:** 10.1038/s41598-018-30760-8

**Published:** 2018-08-17

**Authors:** Robin Mesnage, George Renney, Gilles-Eric Séralini, Malcolm Ward, Michael N. Antoniou

**Affiliations:** 1 Gene Expression and Therapy Group, King’s College London, Faculty of Life Sciences & Medicine, Department of Medical and Molecular Genetics, 8th Floor, Tower Wing, Guy’s Hospital, Great Maze Pond, London, SE1 9RT United Kingdom; 2 Proteomics Facility, King’s College London, Institute of Psychiatry, London, SE5 8AF United Kingdom; 3 University of Caen, Institute of Biology, EA 2608 and Risk Pole, MRSH-CNRS, Esplanade de la Paix, University of Caen, Caen, 14032 Cedex France

Correction to: *Scientific Reports* 10.1038/srep39328, published online 09 January 2017

This Article contains errors.

The Authors neglected to include the data for month 2 (M2) and month 24 (M24) in Figure 1. The correct version of this figure is included below as Figure 1.

**Figure 1:**
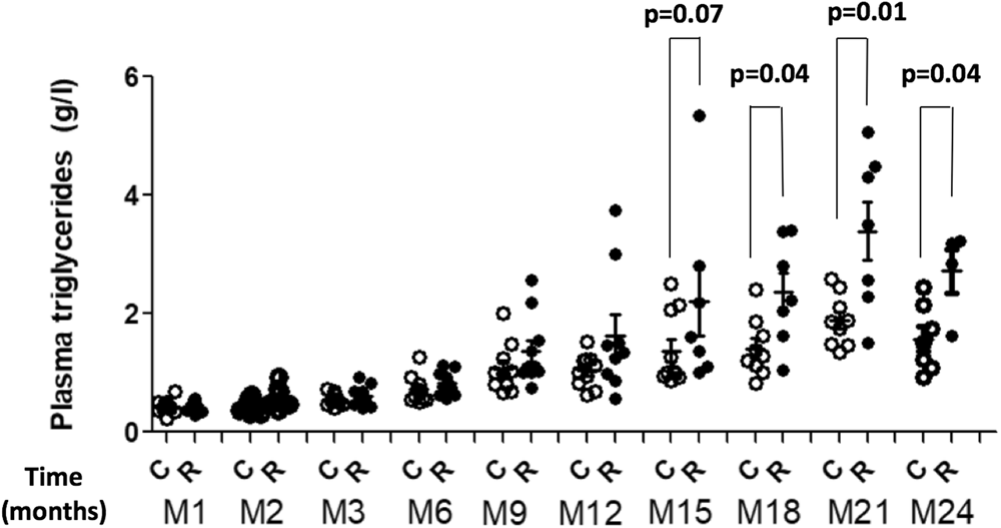
Increased plasma triglyceride levels in rats administered with Roundup. Blood samples was collected from control and Roundup-treated (50 ng/L glyphosate equivalent dilution) female rats following two years exposure via drinking water. Blood was taken via the tail vein of each animal after 1, 2, 3, 6, 9, 12, 15, 18, 21 and 24 months of treatment. Pair-wise comparisons were performed with a Mann-Whitney U test.

Additionally, in the Methods section under the subheading ‘Proteome profiling using Tandem Mass Tag-LC-MS/MS’,

“Precursor mass tolerance for the searches was set at 20ppm and fragment mass tolerance at 0.8ppm.”

should read:

“Precursor mass tolerance for the searches was set at 20ppm and fragment mass tolerance at 0.8 Daltons.”

The Data availability section was omitted from the Additional Information section:

Data availability: The mass spectrometry proteomics data have been deposited to the ProteomeXchange Consortium via the PRIDE [1] partner repository with the dataset identifier PXD010181.

Finally, cross-referencing of the animals’ tumour and age data reported in this Article and References [18] and [29] is described in the Table 1 included below.

**Table 1:** Collation of age and mammary tumour incidence metadata of female rats chronically exposed to Roundup herbicide.

ID	ID-2	TMT	Batch	Age	Mammary tumor
8771	C4	128_N	TMT2	720	0
8787	C3	127_C	TMT1	730	1
8788	R1	129_N	TMT1	701	1
8789	C2	127_N	TMT1	540	0
8820	C5	128_C	TMT2	729	1
8826	R2	129_C	TMT1	723	1
8828	R3	130_N	TMT1	345	1
8837	C1	126	TMT1	646	1
8868	R4	130_C	TMT1	680	1
9055	C1	126	TMT2	728	0
9062	C2	127_N	TMT2	737	1
9147	C3	127_C	TMT2	736	1
9150	R5	131	TMT1	731	0
9151	R1	129_N	TMT2	680	1
9274	R2	129_C	TMT2	738	1
9282	C5	128_C	TMT1	721	0
9313	C4	128_N	TMT1	722	0
9325	R3	130_N	TMT2	505	1
9338	R4	130_C	TMT2	533	1
9512	R5	131	TMT2	715	1

Column ID refers to the identification number of animals in Reference 29. ID-2, TMT and Batch refers to the same animal as described in this Article. Mammary tumor incidence is from Reference 18.

These changes do not affect the conclusions of the Article. The Authors apologise for the errors.

