# Supplementary material for: Multiomics reveal non-alcoholic fatty liver disease in rats following chronic exposure to an ultra-low dose of Roundup herbicide
Source: Sci Rep. 2017 Jan 9;7:39328. doi: 10.1038/srep39328 (PMC5220358; doi:10.1038/srep39328)
Supplement: Supplementary Data Legends [file srep39328-s1.doc]

**Multiomics reveal non-alcoholic fatty liver disease in rats following chronic exposure to an ultra-low dose of Roundup herbicide**

Robin Mesnage^1^, George Renney^2^, Gilles-Eric Séralini^3^, Malcolm Ward^2^ and Michael N Antoniou^1*^

^1^ Gene Expression and Therapy Group, King's College London, Faculty of Life Sciences & Medicine, Department of Medical and Molecular Genetics, 8th Floor, Tower Wing, Guy's Hospital, Great Maze Pond, London SE1 9RT, United Kingdom.

^2^ Proteomics Facility, King's College London, Institute of Psychiatry, London SE5 8AF, United Kingdom.

^3^ University of Caen, Institute of Biology, EA 2608 and Risk Pole, MRSH-CNRS, Esplanade de la Paix, University of Caen, Caen 14032, Cedex, France

^*^ Correspondence: [michael.antoniou@kcl.ac.uk](mailto:michael.antoniou@kcl.ac.uk)

**Additional files**

**Supplementary Table 1. Raw Data of the proteome analysis for the TMT1 exported data.** Raw peak intensity values are indicated for each of the 6358 peptides successfully detected in the TMT1 experiment.

**Supplementary Table 2. Raw Data of the proteome analysis for the TMT2 exported data.** Raw peak intensity values are indicated for each of the 5998 peptides successfully detected in the TMT2 experiment.

**Supplementary Table 3. Proteome data analysis**. Only peptides with TMT reporter ion signal intensities for all ten samples were used for further bioinformatics analysis. Any duplicate peptides were removed before the data was SumScale normalised. The two normalised data files were then merged together to give one 10vs10 file comparison. Any peptides which did not have intensity values in all twenty TMT reporter ion channels were filtered out and median values were taken of the control and treated samples respectively.A total of 1906 peptides were quantified across all liver samples. Pairwise non-parametric Mann–Whitney U tests were performed and a p-value was attributed to each of the 1906 peptides s. The resulting p-values were adjusted by the Benjamini-Hochberg multi-test adjustment method for the high number of comparisons.

**Supplementary Table 4: Proteome verification using Tandem Mass Tag - selected reaction monitoring experiment.** A. Optimised TMT-SRM method to verify selected target peptides. 1 = n fourth transition ion was present for this peptide; 2 = peptides discarded from SRM analysis due to technical repeat CV values being above the 15% threshold. B.

**Supplementary Table 5: annotation analysis of liver genes using the DAVID tool**. The rat genome was used as a background list to calculate the p-values of each term. A total of 131 genes were recognised. The p-values were calculated according to a modified Fisher’s exact test (EASE score) and adjusted according to the Benjamini-Hochberg method. The fold enrichment (FE) of the statistically most overrepresented (enriched) GO biological process and KEGG pathways terms are presented.

**Supplementary Table 6. Raw Data of the metabolome analysis.** Raw peak intensity values are indicated for each of the 673 metabolites successfully detected.

**Supplementary Table 7. Metabolome analysis.** Raw data was extracted, peak-identified and QC processed using Metabolon’s hardware and software. Raw data is available as Supplementary Table 6. Metabolites were identified by automated comparison of the ion features in the experimental samples against a reference library of more than 3000 purified standard compounds that included retention time/index (RI), mass to charge ratio (m/z), and chromatographic data (including MS/MS spectral data), and then curated by visual inspection for quality control using software developed at Metabolon 43. Peaks were quantified using area-under-the-curve. A total of 673 metabolites were detected. The maximum percent missing data allowed was 20%. As a result, 602 peptides were taken forward for bioanalytical analysis. Pairwise non-parametric Mann–Whitney U tests were performed and a p-value was attributed to each of the 602 metabolites. The resulting p-values were adjusted by the Benjamini-Hochberg multi-test adjustment method for the high number of comparisons.

**Supplementary Data S8. Metabolome analysis.** Biochemical classification of the metabolites detected in the metabolome experiment
